# Increase in serogroup W invasive meningococcal disease in England associated with pilgrimage to Saudi Arabia, January 2024 to June 2025

**DOI:** 10.2807/1560-7917.ES.2025.30.31.2500509

**Published:** 2025-08-07

**Authors:** Helen Campbell, Jay Lucidarme, Stephen A Clark, Emma J Heymer, Sonia Ribeiro, Xilian Bai, Shazaad Ahmad, Mary E Ramsay, Ray Borrow, Shamez N Ladhani

**Affiliations:** 1 Immunisation and Vaccine Preventable Diseases Division, UK Health Security Agency, Colindale, London, United Kingdom; 2 Meningococcal Reference Unit, UK Health Security Agency, Manchester Royal Infirmary, Manchester, United Kingdom; 3 Centre for Neonatal and Paediatric Infections (CNPI), St. George’s University of London (SGUL), London, United Kingdom

**Keywords:** Mass gatherings, Meningococcal disease, Public health, Travel, Vaccine, MenW

## Abstract

England is experiencing an increase in serogroup W invasive meningococcal disease (IMD) caused by ‘strain A’ of the meningococcal sequence type 11 clonal complex (MenW:cc11) Hajj strain sublineage. Travel-associated and non-travel-associated meningococci from four strain A substrains (A1–A4) accounted for 32 of 59 MenW IMD cases between January 2024 and June 2025, 14 in people returning from Saudi Arabia or household contacts; eight of 14 linked to Umrah pilgrimage. Communications about MenACWY vaccination for pilgrims year-round should be reinforced.

In the lead up to the Hajj 2024 (taking place 14–19 June), England was part of an international cluster of meningococcal serogroup W (MenW) outbreaks associated with travel to Saudi Arabia for Umrah as well as travel to/from other Middle Eastern and Asian countries, and among Muslim community members without known travel links [3,4]. Meningococci from 29 invasive meningococcal disease (IMD) and one conjunctivitis case from various countries belonged to one of five distinct phylogenetic clusters (substrains) within two broader strains (strains A and C) of the MenW ST-11 complex Hajj strain sublineage. Travel to Saudi Arabia was reported for two of three cases of the single strain C substrain, for six of eight cases of strain A substrain A2 and for five of eight cases of strain A substrain A3. Other travel destinations for substrain A2 were Kenya via Turkey (n = 1) and Mauritius (n = 1), and India for substrain A3 (n = 1). Strain A substrain A1 was associated with travel to Egypt and Abu Dhabi (1/9 cases), and strain A substrain A4 with travel to the United Arab Emirates (2/2 cases).

In England, cases continued to increase after the initial outbreak report, with 59 MenW IMD cases confirmed between January 2024 and June 2025. Here we describe these recent cases, including epidemiological links, recent travel and outcomes.

## Diagnosis and confirmation - meningococci

**Figure 1:**
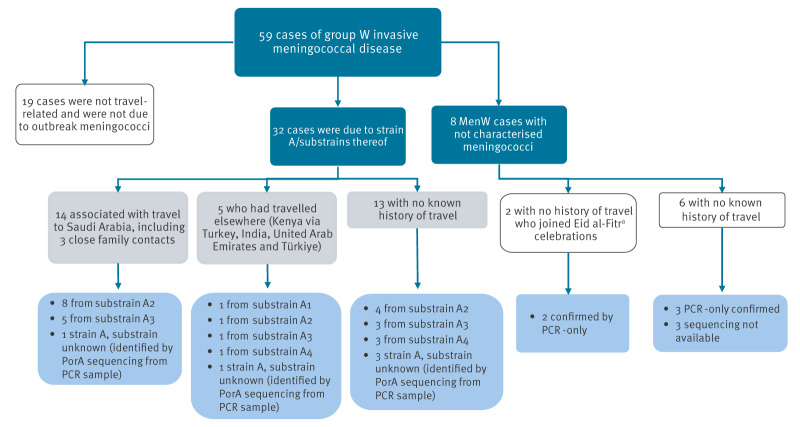
Classification and characterisation of group W invasive meningococcal disease cases, England, January 2024–June 2025 (n = 59)

^a^ An Islamic festival celebrated worldwide by Muslims.

**Figure 2:**
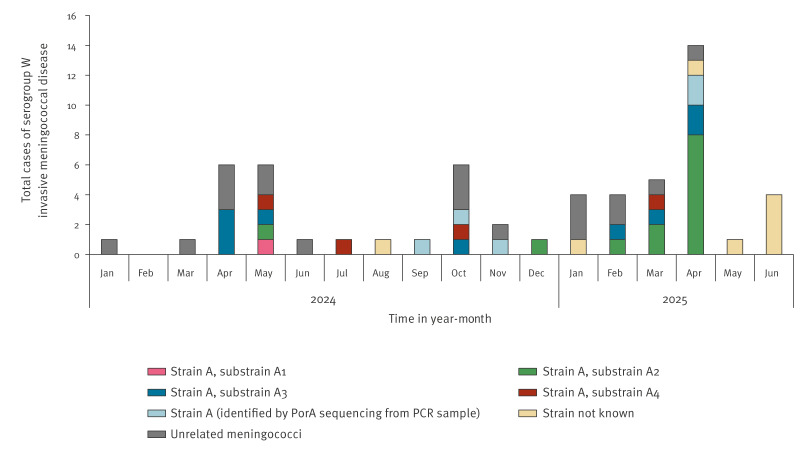
Temporal distribution of serogroup W invasive meningococcal disease cases, by month of confirmation and meningococcal strain characteristics, England, January 2024–June 2025 (n = 59)

Of the 59 MenW IMD cases confirmed over the 18-month period, 32 were caused by strain A [5]. Of these, 27 were culture-confirmed and identified by genome sequence analysis as belonging to strain A substrains A1 to A4. Among these, substrains A2 and A3, with links to Saudi Arabia, predominated in 2025 (Figure 1, Figure 2). Five cases confirmed by PCR only were identified as belonging to strain A based on the presence of NEIS1364 (PorA) allele 1091, a strong marker for strain A (personal communication: Muhamed-Kheir Taha, Pasteur Institute, 15 April 2025). No non-culture marker yet exists to distinguish between the four strain A substrains. Nineteen cases were confirmed not to be due to strain A, nor were they linked to travel (Figure 1). Of the remaining eight as yet uncharacterised meningococci, two had recently travelled to Saudi Arabia. In addition to the 32 cases due to strain A, there was a non-invasive conjunctivitis case due to substrain A3 in a child with no travel links in 2024.

None of the 32 cases due to strain A were reported to have received the MenACWY vaccine. All 27 strain A cultures were sensitive to penicillin, rifampicin, ciprofloxacin and cefotaxime (data not shown).

## Epidemiology and clinical follow-up

**Figure 3:**
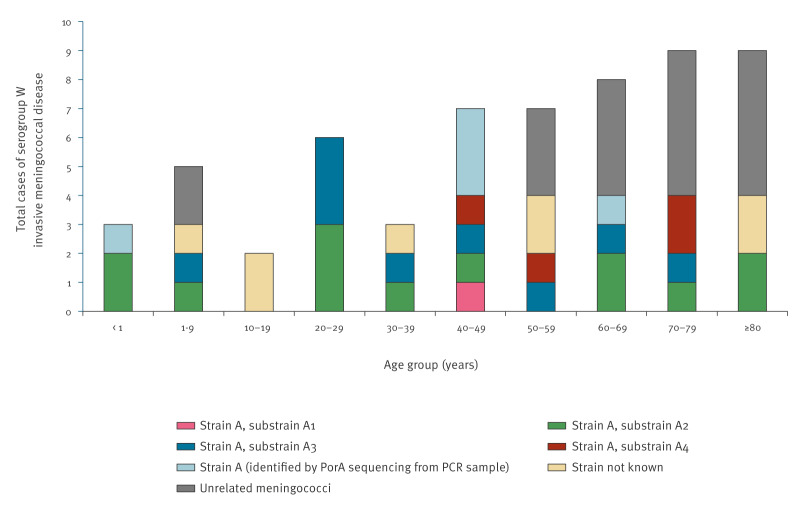
Confirmed cases of serogroup W invasive meningococcal disease, by age group and meningococcal characteristics, England, January 2024–June 2025 (n = 59)

The 32 strain A IMD cases occurred across all age groups (median age: 43 years, range: 0–87 years) with a case fatality rate of 9% (3/32), and two travel-associated cases aged 10–19 years were caused by as-yet uncharacterised meningococci. In contrast, the 19 MenW IMD cases caused by unrelated (non-strain A) meningococci occurred predominantly in ≥ 50-year-olds (17/19 cases; median age: 71 years; range: 2–89 years) with a case fatality rate of 16% (3/19); all deaths occurred among ≥ 80-year-olds (Figure 3).

Of the 59 total cases, most (n = 45) were confirmed in blood, eight in cerebrospinal fluid and six from another site (three joint, two pericardial samples and one pleural fluid). The IMD cases caused by strain A resided in different locations across four Regions of England (London, Midlands and East of England, North of England and South of England). Patient addresses did not indicate close geographical clustering other than two sets of two cases living within 2 km of each other. Four cases caused by strain A would have been eligible for the teenage MenACWY vaccine but none of these, nor anyone who was a known Umrah pilgrim and thus should have been vaccinated, were documented as MenACWY-vaccinated. A similar proportion of cases arose in males and females overall, with 31 and 28 cases respectively, while 19 of the 32 IMD caused by strain A occurred in males.

## Discussion

In England, MenW IMD is rare because of effective direct and indirect (population) protection, afforded by the national adolescent MenACWY immunisation programme introduced in 2015. During COVID-19 pandemic restrictions, IMD cases due to all serogroups declined but, after restrictions were lifted in July 2021, serogroup B cases increased while cases due to serogroups C, W and Y (serogroup A is very rare in England) remained low [1]. Modelling predicts long-lasting reductions in post-pandemic carriage and disease due to serogroups C, W and Y [2].

Large congregations increase the risk of meningococcal transmission, which can lead to extended IMD outbreaks and global spread. In particular, the religious pilgrimage to Mecca in Saudi Arabia for Hajj (able-bodied adult Muslims are required to perform pilgrimage at least once in their lifetime during the 12th month of the Islamic Calendar) or Umrah (a shorter non-compulsory year-round Muslim pilgrimage) involves millions of international pilgrims and has been associated with several large international IMD outbreaks since the 1980s. This led to the Saudi Ministry of Health to mandate MenACWY vaccination for all pilgrims attending Hajj and Umrah [5], thereby markedly reducing the incidence of pilgrimage-associated IMD until recently [6].

Following the early 2024 increase in MenW cases, the UK Health Protection Agency (UKHSA) and the UK National Travel Health Network and Centre (NathNAC) reinforced recommendations for MenACWY vaccination for pilgrims entering Saudi Arabia and for pilgrims to seek immediate medical attention if they developed symptoms or signs of IMD. Cases of strain A that arose in contacts of Saudi Arabian travellers and in the Muslim community following Eid al-Fitr celebrations (an Islamic festival celebrated worldwide by Muslims) further highlight the importance of protecting others through use of MenACWY conjugate vaccine ahead of travel. The increase in MenW IMD in England coincided with Ramadan, when there is intensified Umrah pilgrimage, while the reductions in cases during and just after Hajj in June 2024 and again during and just after Hajj in June 2025 are probably due to increased enforcement of MenACWY vaccination during Hajj compared with the rest of the year [7].

Here we show that imported invasive meningococcal strains linked to Umrah in England during 2024 and 2025 belonged to four distinct substrains of strain A of the MenW:cc11 Hajj strain sublineage. This is consistent with the observation of multiple substrain expansions occurring in parallel in early 2024 [5]. Interestingly, there appeared to be a shift in substrain dynamics from early 2025, with substrains A2 and A3 predominating. This could represent natural fluctuations in substrain incidences or perhaps indicate a permanent shift in the predominance of particular substrains, e.g. in terms of geographical distribution, carriage rates and virulence.

All strain A isolates were susceptible to commonly used antimicrobials. This is reassuring given that resistance to ciprofloxacin (the first-choice antibiotic for chemoprophylaxis) has been reported in diverse carriage strains among Hajj pilgrims [8] as well as the distinct W:cc11 Hajj strain sublineage strain C substrain isolated in other countries during the 2024 outbreak (n = 3). These reports raise concerns about the potential for wider transmission of ciprofloxacin-resistant meningococci globally through pilgrims [4]. In November 2024, UK public health guidance was updated to consider rifampicin chemoprophylaxis for close contacts of suspected and confirmed IMD cases recently returned from the Middle East (including Saudi Arabia) or Asia, unless the infecting strain is known to be ciprofloxacin sensitive [9].

## Conclusions

The increase in MenW IMD cases among pilgrims returning from Saudi Arabia is concerning because of the severity of MenW disease and the potential for endemic transmission. This risk is not restricted to the UK but also applies to many other countries worldwide with pilgrims attending Umrah and Hajj every year. There is an urgent need to reinforce communications for MenACWY vaccination for all pilgrims to Saudi Arabia, not only for Hajj but also for Umrah, as well as increased vigilance of IMD among returning pilgrims with the potential for spread to their households and the local community.

## Data Availability

Draft genomes generated in connection with this investigation are available on the PubMLST Neisseria database (https://pubmlst.org/organisms/neisseria-spp ) under the following accession numbers: 150006, 150007, 150009, 150199, 150200, 150202, 151144, 162389, 162407, 162547, 166206, 168556, 168564, 168566, 168567, 168572, 168582, 169255, 169256, 169257, 169260, 169271, 169272, 169275, 169278, 169281, 169282.
